# Supplementary material for: Prognostic Value of Pulmonary Artery Pulsatility Index in Right Ventricle Failure-Related Mortality in Inoperable Chronic Thromboembolic Pulmonary Hypertension
Source: J Clin Med. 2022 May 12;11(10):2735. doi: 10.3390/jcm11102735 (PMC9147458; doi:10.3390/jcm11102735)
Supplement: Supplementary file 1 [file jcm-11-02735-s001.zip › jcm-1676431-supplementary.pdf]

Supplementary Table S1. Characteristics of inoperable CTEPH patients excluded from the study.

| Patient ID | Sex | Age at diagnosis (years) | Cause of exclusion | Cause of death |
|---|---|---|---|---|
| 1 | F | 70 | Lack of consent | |
| 2 | M | 83 | Non-CTEPH related death | Colon cancer |
| 3 | F | 67 | Non-CTEPH related death | Abdominal surgery-related complications |
| 4 | F | 89 | Non-CTEPH related death | Sepsis |
| 5 | F | 27 | CTEPH persistent after PEA | - |
| 6 | F | 57 | CTEPH persistent after PEA | |
| 7 | M | 73 | Non-CTEPH related death | Orthopedic surgery related complication |
| 8 | F | 36 | Lack of follow-up | |
| 9 | M | 66 | Lack of consent | |
| 10 | F | 78 | Lack of follow-up | |
| 11 | M | 59 | CTEPH persistent after PEA | |
| 12 | M | 67 | CTEPH persistent after PEA | |
| 13 | F | 70 | Lack of consent | |
| 14 | M | 53 | Lack of consent | |
| 15 | M | 55 | CTEPH persistent after PEA | |
| 16 | M | 89 | Non-CTEPH related death | COVID-19 |
| 17 | M | 70 | Non-CTEPH related death | Sepsis |
| 18 | F | 74 | Non-CTEPH related death | Sepsis |
| 19 | M | 59 | Non-CTEPH related death | Colon cancer |
| 20 | F | 61 | Unknown cause of death | |

Abbreviations: CTEPH – chronic thromboembolic pulmonary hypertension; F – female; M – male; PEA – pulmonary endarterectomy
